# Impact of stocking density on beef heifer productive performance and health through hematology and *longissimus dorsi* ultrasound

**DOI:** 10.3389/fvets.2026.1826548

**Published:** 2026-06-03

**Authors:** Anastasia Lisuzzo, Giorgia Taio, Silvia Miretti, Carmine Versace, Paolo Cornale, Antonio Mimosi, Enrico Fiore, Francesca Cecchini, Chiara Tommasoni, Matteo Boso, Matteo Gianesella

**Affiliations:** 1 Department of Animal Medicine, Production and Health (MAPS), University of Padua, Legnaro, Italy; 2 Department of Veterinary Sciences, University of Turin, Grugliasco, Italy; 3 Department of Agricultural, Forest and Food Sciences, University of Turin, Grugliasco, Italy; 4 Veterinary Practitioners, Eraclea, Italy

**Keywords:** animal growth, cattle, muscle ultrasound, space allowance, stress

## Abstract

Stocking density is one factor that can stimulate stress conditions in beef cattle farming, negatively impacting animal health and performance. The aim of this study was to evaluate hematology and *longissimus dorsi* ultrasound in beef heifers housed at two different stocking densities. A total of 54 Charolais heifers, 27 animals in each trial for two consecutive years, were randomly divided into two groups at arrival: high stocking density group (HIGH_D, 5.54 m^2^/animal; *N* = 26), and low stocking density group (LOW_D, 10.3 m^2^/animal; *N* = 28). A longitudinal study design was applied and animals were evaluated with blood sampling and *longissimus dorsi* ultrasound at arrival (D0), and after 30, 60, 90 and 120 days (D30, D60, D90, and D120) until the slaughtering. Body weight was measured at D0, D60, and D120 to calculate average daily gain (ADG) for the periods D60-D0, D120-D60, and D120-D0. Finally, the carcass yield and quality were obtained at the slaughterhouse. Statistical analysis was conducted with linear mixed models. The LOW_D had a greater body weight at D120, as well as higher ADGD120_D0, ADGD120_D60, and carcass yield compared to HIGH_D. The same group had also lower neutrophils and greater lymphocytes between D60 and D120, and lower monocytes between D90 and D120. Instead, HIGH_D had an increase in neutrophils and monocytes, and a decrease in lymphocytes. Despite both groups were within physiological ranges, the HIGH_D condition could be related to a slightly stress condition. Moreover, the LOW_D showed a greater muscle thickness and ribeye area from D30, back-fat thickness from D60, and intramuscular fat from D90 until D120. In conclusion, this study confirmed that stocking density significantly affects beef heifer performance and health, with animals housed at 10.3 m^2^/animal having better growth outcomes, ultrasound fat deposition pattern, and muscle growth.

## 1 Introduction

Beef cattle farming is particularly important in Italy, which is the fifth largest contributor to European production (1). The Italian system is mainly based on fattening young cattle, of which 45% is imported from France, Ireland, and Eastern European countries. The Charolais breed is among the most commonly used imported breeds for this type of farming (2). Growing public interest in animal welfare, including beef cattle, is driving changes the livestock industry to increase production efficiency while promoting animal welfare (3). Animal welfare is generally referred to animal’s ability to adapt to an environment. When an animal is unable to adapt, or when the adaptation requires a great deal of time and energy, welfare is considered poor (4). Adaptations can include behavioral responses, performance (e.g., reduced growth), and immune activity. Changes in the growth represent the most economical responses, while alterations in behavior and immune activity are associated with a deterioration in animal well-being (4, 5). Among immune activity parameters, hematological profile is the primary determinant of animals’ adaptation to their environment and, in turn, their welfare (6). In fact, this animal-based measure is one of the most commonly used indicators for monitoring stress in both acute and chronic situations in both beef and dairy cattle (5, 6). Stocking density and animals’ group size are factors that can stimulate acute and/or chronic stress in beef cattle farming (3, 7). In fact, these factors affect the animals’ ability to access resources, rest comfortably, regulate their body temperature, and interact socially or avoid negative interactions (4). Consequently, animal behavior, welfare, growth, and health can be affected by these conditions (2, 8).

One method for evaluating *in vivo* the performance of beef cattle is the *longissimus dorsi* ultrasound, which assesses both the fat and muscle components. This technique is based on measurements of the thickness of the fat cover (back-fat thickness), muscle thickness, ribeye area, and muscle ultrasound texture also called intramuscular fat or ultrasound marbling (9). In fact, ultrasound echoes are reflected by the fat-muscle interface. Based on the intensity and distribution of the returning echoes, it is possible to make an appropriate assessment of muscle marbling in live animals (10). The marbling or intramuscular fat content is considered one of the main factors in assessing meat quality, particularly the tenderness, juiciness, and aroma (11, 12). This type of fat is located between and within muscle fibers with the greatest accumulation in the final stages of the growth process. Furthermore, the estimated intramuscular fat by ultrasound had a good correlation with meat fat, about 76–85% (12, 13). Its evaluation can be performed at the dorsal level between the 12th and 13th ribs for better correlation with carcass characteristics as well as for the evaluation of back-fat thickness and ribeye area (14, 15).

Currently, there are no European regulations establishing the minimum space allowance for beef cattle older than 6 months (2, 16). Furthermore, most studies in the literature have focused on a stocking density of less than 3.0 m^2^/animal, although there are indications that this space allowance should be increased (5). In fact, recommendations in the EU literature for commercial beef cattle farms range from 2.4 to 5.5 m^2^/animal for pens with bedding, depending on animal weight. Nevertheless, a minimum space allowance of 13 m^2^/animal is suggested for animals weighing more than 400 kg, to allow synchronized lying down and further reduce group stress. Despite this recommendation, few studies have focused on stocking densities above 6 m^2^/animal (16). These studies would also be necessary to understand when increasing the available space no longer brings additional benefits (8). Moreover, this increase in space allowance would have a significant impact on farms’ sustainability, as the number of animals raised in current facilities would need to be reduced or, alternatively, additional housing facilities would be required (5).

The hypothesis of this study was that increasing space allowance above 6 m^2^/animal would have a positive effect on beef cattle health and performance. For this reason, the aim of the study was to evaluate hematology and *longissimus dorsi* ultrasound in beef heifers housed at 5.5 or 10.3 m^2^/animal.

## 2 Materials and methods

### 2.1 Ethical statement

Animal care and procedures were conducted in accordance with the Guide for the Care and Use of Laboratory Animals and Directive 2010/63/EU for animal experiments (National law: D. L. 26/2014). The Ethics Statement was approved by the Animal Care and Use Committee of the University of Padua (protocol number 17498/2024).

### 2.2 Animal and study design

The study was conducted in one commercial beef cattle barn located in Veneto (Italy) over two subsequent years, with the same season effect (March to July 2024 and March to July 2025). All animals were fed the same total mixed ratio (Table 1) twice daily based on 10% feed refusal, and water was provided *ad-libitum*. The farm used indoor boxes of 6 × 12 m, where 6 m represented the linear feed space, and straw bedding.

**Table 1:** Feed ingredients (Kg/animal) and analytical components (dry matter basis) of the diet used for all animals during the trial.

Ingredients	Kg / animal
Fat prest cereal premix	7.5
Whole corn mash	4.0
Protein, vitamin and mineral premix	3.0
Maize meal	1.0
Sugar beet pulps	0.5
Wheat straw	0.3
Parameter	% Dry matter*
Crude protein	14.84
Neutral Detergent Fiber (NDF)	33.10
Starch	32.58
Ether extract (EE)	3.41
Calcium	1.04
Phosphorous	0.48

*Dry matter value was 49.61%.

A total of 54 Charolais heifers divided into experimental trials, one per year of 27 animals each, were enrolled in this study with an average age of 13.2 ± 1.2 months at arrival. Upon arrival each trial, animals were randomly allocated to one of two stocking density treatments: a high stocking density group (HIGH_D), reflecting the routine housing conditions adopted on the farm, and a low stocking density group (LOW_D), representing a compromise between animal welfare and farm management requirements.

All animals were housed in indoor boxes measuring 6 × 12 m. Across the two trial, the LOW_D treatment included 28 heifers housed in four boxes (two per trial), with seven animals per box, providing 10.3 m^2^ per animal. The HIGH_D treatment included 26 heifers housed in two boxes (one per trial), with 13 animals per box, providing 5.54 m^2^ per animal.

**Figure 1:**
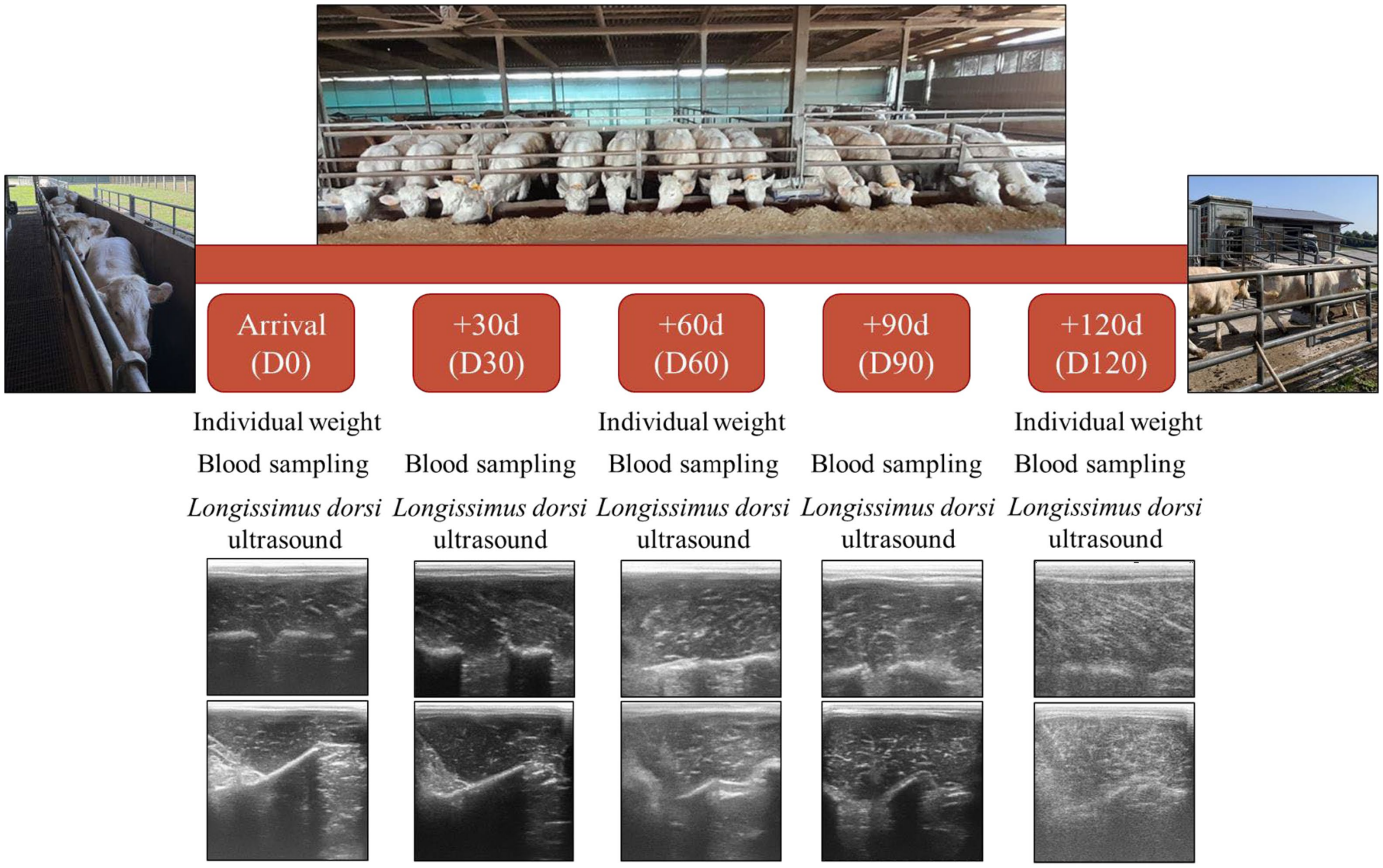
Longitudinal study design.

A longitudinal study design was applied in the trial (Figure 1). Blood sampling and *longissimus dorsi* ultrasound evaluations were performed at arrival (D0), and after 30, 60, 90 and 120 days (D30, D60, D90, and D120) until the slaughtering. The animal body weight (BW; kg) was measured at D0 (BWD0), D60 (BWD60), and D120 (BWD120). The average daily gain (ADG; kg/day) was then calculated between D60 and D0 (ADGD60_D0), D120 and D60 (ADGD120_D60), and D120 and D0 (ADGD120_D0). Finally, the carcass yield (ratio between carcass weight and live weight), and quality according to S-EUROP classification (17) were obtained from the slaughterhouse according to national and European regulations.

### 2.3 Blood sampling and analysis

Blood samples were obtained from the jugular vein using evacuated tubes and stored in tube containing EDTA (9 mL; Terumo Venoject, Leuven, Belgium). The samples were then refrigerated at 4 °C and transported in a portable freezer (CoolFreeze CFX65 W professional, Dometic, Stockholm, Sweden; minimum temperature −22 °C) at the same constant temperature to the laboratory of the Department of Animal Medicine, Production and Health (MAPS) of the University of Padua within 3 h to perform complete blood count analysis.

The hematological automatic analyzer (ADVIA 120; Siemens, Padova, Italy) was used to perform a complete blood count analysis to determine the red blood cell (×10^6^ cells/μL; RBC), hemoglobin (g/dL; HGB), hematocrit (%; HCT), medium corpuscular volume (fL; MCV), mean corpuscular hemoglobin concentration (g/dL; MCHC), platelets (×10^3^ cells/μL; PLT), white blood cell (×10^3^ cells/μL; WBC), neutrophils (%; NEU), lymphocytes (%; LYM), monocytes (%; MON), eosinophils (%; EOS), basophils (%; BAS), and large unstained cells (%; LUC).

### 2.4 The *longissimus dorsi* ultrasound evaluations

The *longissimus dorsi* ultrasonography was performed on the right side of each animal between the 12th and 13th rib (12). Animals were not clipped and ethyl alcohol (90%) was used as a transducing agent. The portable ultrasound scanner (Draminski® Ultrasound Scanner Blue, Draminski® S. A., Olsztyn, Poland) and multifrequency linear probe were used for the examination. All scans were performed by a trained veterinarian using a constant setting using a frequency of 5.0 MHz, 15 cm depth acoustics window, 100% gray scale gain, and time-gain compensation was in a neutral position. Images were saved in JPEG format. The saved ultrasound images were used for a post sampling measurement using Image-J software (Wayne Rasband, Rockville Pike, Bethesda, United States). Two scans were performed for each evaluation, one cross-sectional and one longitudinal image. The cross-sectional image was used to measure the ribeye area (RIBEYE; cm^2^), while the longitudinal was used for back-fat thickness (BFT; cm), muscle depth (DEPTH; cm), and estimated percentage of intramuscular fat (IMF; %) (9). The IMF was estimated using the formula of the texture analysis proposed by (12) on living beef cattle.

### 2.5 Statistical analysis

The minimum sample size was calculated *a priori* using G*Power software v.3.1.9.7.^1^ This analysis used the values of ADG as a discriminant between stocking density presented by Marquis et al. (1991) using an *α* of 0.05, an *β* of 0.05, and an effect size of 2.60. Consequently, the total minimum sample size was 9 animals.

Differences in carcass quality (S-EUROP classification; %) were evaluated with the Chi-square test by the MedCalc software ver. 19.4 (MedCalc Software, Ostend, Belgium).

All other statistical analysis was performed with R software v.4.2.3.^2^ Data normality was confirmed using the Shapiro–Wilk test (Supplementary Table 1). The Akaike information criterion (AIC) and the Bayesian information criterion (BIC) to select the model based on performance (lowest AIC and BIC). The BWD0, BWD60, BWD120, ADGD60_D0, ADGD120_D60, ADGD120_D0, and the carcass yield were analyzed using a linear mixed model included the fixed effect of group (2 levels: LOW_D and HIGH_D), boxes (6 levels; boxes 1 to 3 for year 1, and boxes 4 to 6 for year 2), and their interactions. Animal was used as a random effect, while BW at arrival (D0) was used as covariate. A second model was used to evaluated hematological and ultrasound parameters with the fixed effect of group (2 levels: LOW_D and HIGH_D), time (5 levels: D0; D30; D60; D90; and D120), boxes (6 levels; boxes 1 to 3 for year 1, and boxes 4 to 6 for year 2), and their interactions. Animal was used as a random and repeated effect, while BW at arrival (D0) was used as covariate. In both cases, a post-hoc pairwise comparison among LSMEANS was performed using Tukey correction. In addition, a post-hoc power analysis was also performed on the significant results to ensure a power equal or greater than 0.80 (Supplementary Table 2).

In general, a *p*-value ≤ 0.05 was used to consider statistically significant differences, while a p-value between 0.05 and 0.10 was considered a trend towards significance.

## 3 Results

No differences were observed between boxes within the same group in all statistical analysis therefore excluding a trial effect.

The general characteristics and growth traits of the animal are presented in Table 2. The BWD0 and BWD60 did not differ between groups. Instead, the LOW_D had a greater BWD120 compared to HIGH_D (*p-*value = 0.007). Similarly, the ADGD60_D0 was not influenced by group effect, while the ADGD120_D60.

**Table 2:** Animal general and growth characteristics (body weight or BW; and average daily gain or ADG) according to stocking density (LOW_D and HIGH_D).

General characteristics	LOW_D	HIGH_D
N	28	26
n. animal per box	7	13
n. replicates	4	2
Stocking density (m^2^/animal)	10.3	5.54
Weight distribution at D0, kg/m^2^	40.7	74.9
Weight distribution at D120, kg/m^2^	57.4	103.8
Coefficient k^*^ at D0	0.184	0.099
Coefficient k^*^ at D120	0.146	0.079

Growth characteristics	LOW_D	HIGH_D	SEM	*p-*values
BWD0, kg	418.6	414.7	4.84	0.553
BWD60, kg	506.2	505.3	4.82	0.664
BWD120, kg	590.3	574.9	4.80	0.007
ADGD60_D0, kg/d	1.50	1.50	0.10	0.304
ADGD120_D60, kg/d	1.40	1.13	0.10	<0.001
ADGD120_D0, kg/d	1.43	1.34	0.11	0.073
Carcass yield, %	58.89	57.96	0.01	0.023
S-EUROP quality^**^				
E-2	50.0%	53.8%	\	0.782
E-3	42.9%	38.5%	\	0.745
U-3	7.10%	7.70%	\	0.934

*Inversely measured by the formula m^2^ = k × BW^0.667^ (4, 23). ** E: Excellent conformation class; U: Very Good conformation class; 2: Slight fat cover; 3: Average fat cover 17.

(*p-*value < 0.001) and ADGD120_D0 (*p-*value = 0.073) were greater in LOW_D compared to HIGH_D. In addition, heifers in LOW_D had a greater carcass yield at slaughtering (*p-*value = 0.023).

**Figure 2:**
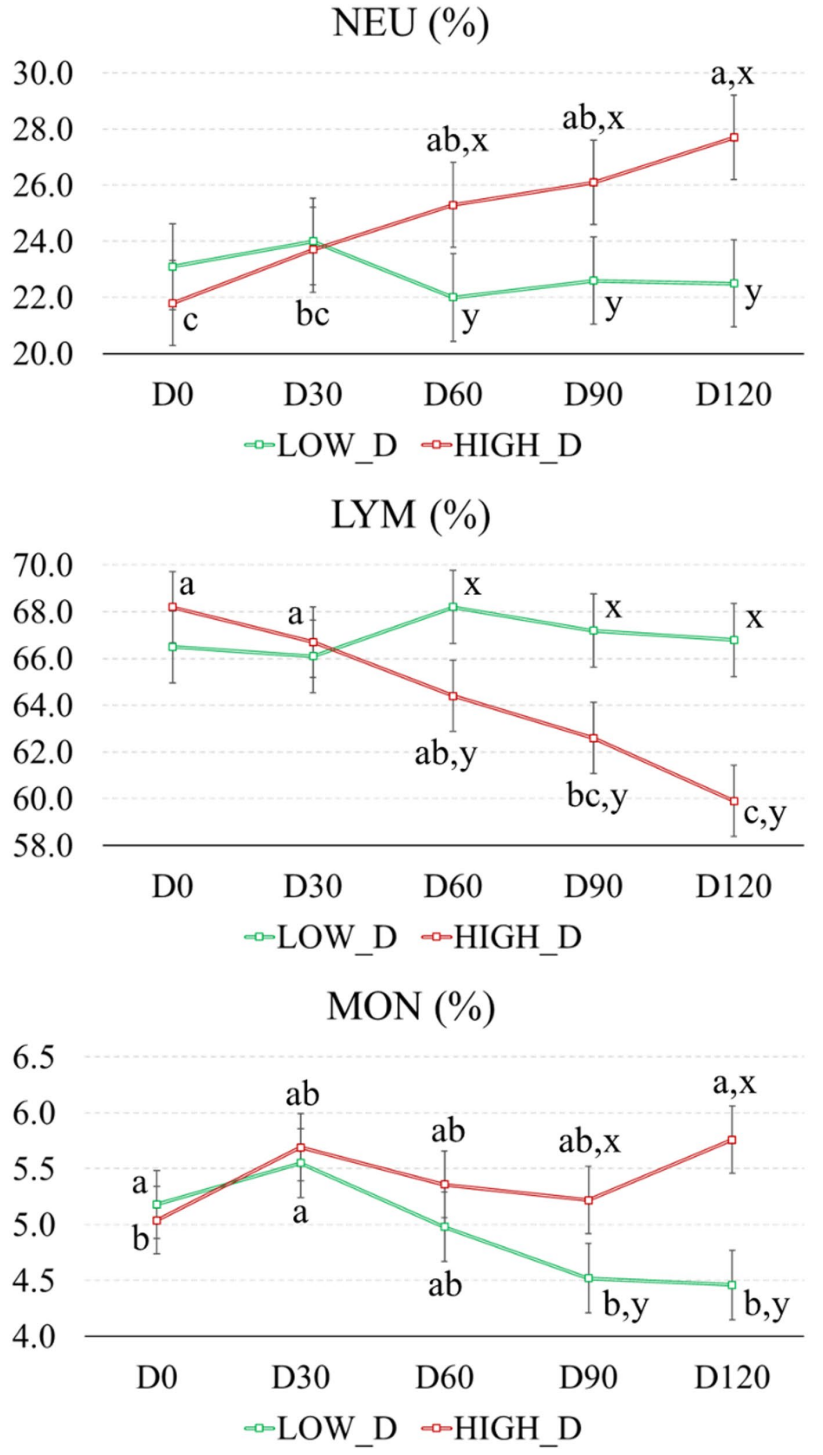
Significant parameters of the complete blood count according to stocking density (LOW_D group, *N* = 28, 10.3 m^2^/animal; HIGH_D group, *N* = 26, 5.54 m^2^/animal), and time (D0, D30, D60, D90, D120). (a–c) Significant differences within group; (x, y) significant differences between groups.

Among the complete blood count parameters, only NEU (*p-*value = 0.012), LYM (*p-*value = 0.008) and MON (*p-*value = 0.031) were different between groups (Figure 2). Specifically, the LOW_D had lower NEU and greater LYM between D60 and D120, and lower MON between D90 and D120 compared to HIGH_D. Moreover, the NEU and LYM of LOW_D heifers did not change over time, while the HIGH_D heifers had a progressive increase in NEU and decrease in LYM from arrival to the slaughtering. Regarding the MON, the LOW_D had a decrease from arrival to the end of the study, while HIGH_D had an opposite trend.

The not significant parameters for group and group*time effects (RBC, HGB, HCT, MCV, MCHC, PLT, WBC, EOS, BAS, and LUC) were presented in Supplementary Table 3. The mean values of this parameters ranged from: 8.57 to 9.80 × 106 cells/μL for RBC; 12.9 to 14.2 g/dL for HGB; 35.5 to 36.9% for HCT; 38.3 to 41.6 fL for MCV; 36.9 to 38.5 g/dL for MCHC; 304 to 356 × 10^3^ cells/μL for PLT; 7.95 to 8.87 × 10^3^ cells/μL for WBC; 2.36 to 4.49% for EOS; 0.96 to 1.18% for BAS; and 0.29 to 0.72% for LUC.

**Figure 3:**
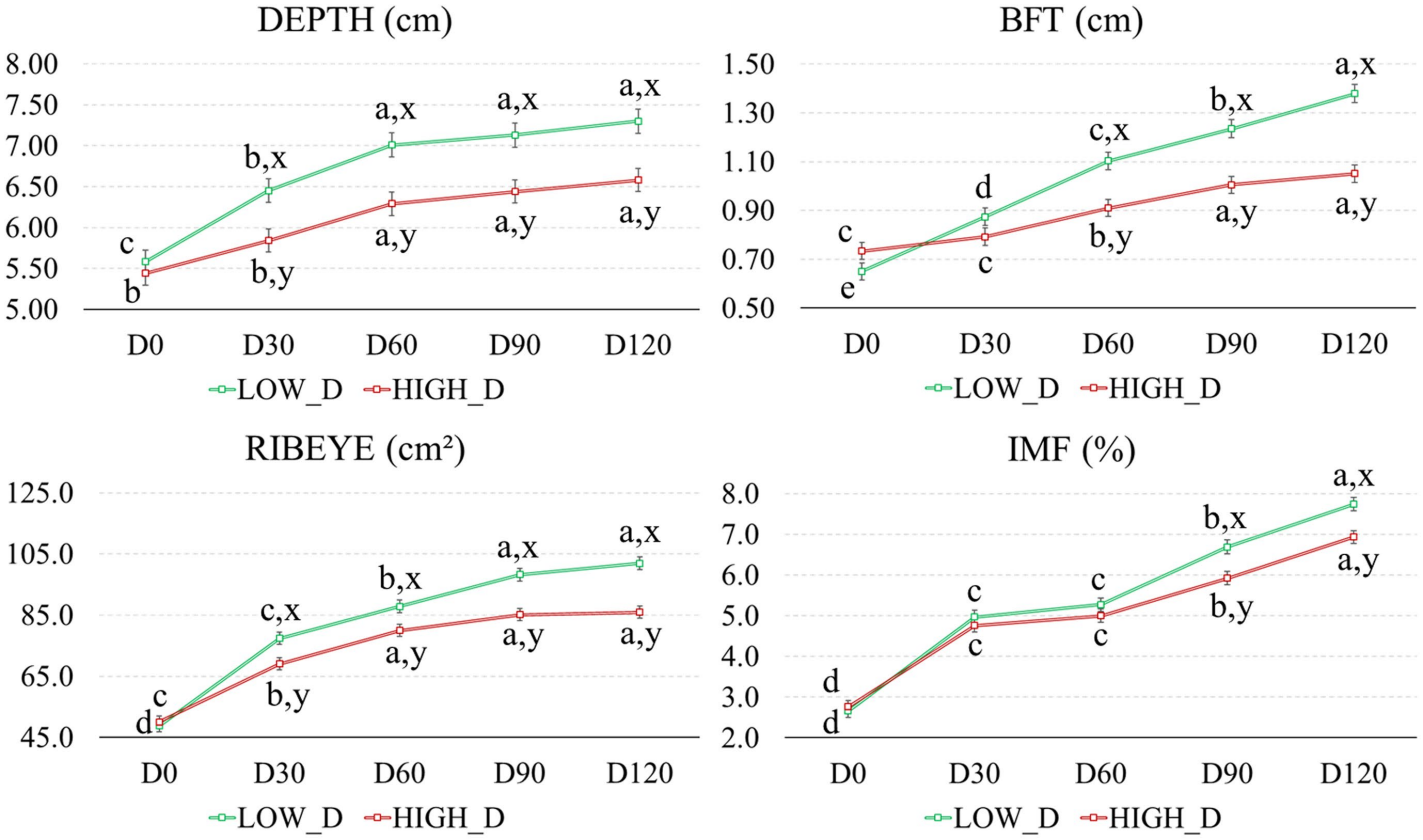
Significant parameters of the *longissimus dorsi* ultrasonography according to stocking density (LOW_D group, *N* = 28, 10.3 m^2^/animal; HIGH_D group, *N* = 26, 5.54 m^2^/animal), and time (D0, D30, D60, D90, D120). (a–d) Significant differences within group; (x, y) significant differences between groups.

All ultrasound parameters displayed a progressive increase from arrival to the end of the study in both groups with differences between groups (Figure 3). Specifically, the DEPTH (*p-*value = 0.021) and RIBEYE (*p-*value < 0.001) were greater in LOW_D compared to HIGH_D heifers from D30 to D120, while the BFT (*p-*value < 0.001) was greater in the same group from D60 to D120, and IMF (*p-*value 184 = 0.015) was greater from D90 to D120.

## 4 Discussion

Stocking density or space allowance is a well-recognized factor affecting both welfare and productivity responses in beef cattle (3). Reduced space allowance has been associated with stress-related conditions leading to decreased dry matter intake and gastrointestinal function (2). Despite its relevance, no specific European legislation currently defines minimum space requirements for cattle old than 6 months (16). For this reason, space recommendations are often derived from kinematic-based models rather than performance outcomes. According to the equation (m^2^/animal = *k* × BW^0.66^) proposed by Mayes et al., a *k* of 0.047 is required to allow the transition between standing and lying, suggesting that both the space allowances adopted in the present study exceeded the minimum threshold for basic postural movements, especially in the LOW_D treatment. However, most available studies have evaluated space allowances below 3.0 m^2^/animal, whereas information on higher space allowances, exceeding 6.0 m^2^/animal, remain limited (5, 16). In this context, the comparison between HIGH_D (5.54 m^2^/animal) and LOW_D (10.3 m^2^/animal) provides novel evidence on the productive responses of beef heifers managed under space allowances that are more representative of welfare- oriented housing conditions.

The effect of stocking density on animal BW or ADG remains debated. Several studies have reported no differences in growth performance across a wide range of space allowances, including comparisons between 1.20 and 2.09 m^2^/animal (*k* = 0.027 to 0.047), between 3.5 and 4.37 m^2^/animal, and even between 14 and 25.000 m^2^/animal (2–4). Conversely, other studies have observed increased BW or ADG with a decreased stocking density, particularly when space allowance increased from approximately 1.3–1.5 to 2.0–2.3 m^2^/animal, and from 2.0 to 3.0 m^2^/animal (18–20). Further improvements in growth performance have been reported up to 4.5–4.7 m^2^/animal, with no additional benefits observed when space allowance was increased to 6.0 m^2^/animal (5, 8, 21). Results are less consistent at higher space allowance (≥10 m^2^/animal), with greater final BW reported at 17 m^2^/animal, but high ADG observed at 10 m^2^/animal (22). Similarly, no difference in BW or ADG were observed when space allowance was expressed using *k* coefficient of 0.033 or 0.048 (23).

The two different stocking density applied in the present study significantly affected both final BW (BWD120) and ADG, with greater values in LOW_D group. Moreover, the ADG differed only after mid-cycle (D60; ADGD120_D60), with heifers housed at lower density exhibiting around 300 gr/d greater ADG than those in HIGH_D. This divergence resulted in a greater overall ADG across the entire production cycle (ADGD120_D0). These findings suggested that the impact of space allowance on growth performance may become more evident as animal increase in size and metabolic demand. Importantly, the *k* coefficients calculated in the present study were substantially higher than the minimum threshold proposed for postural transitions, even at the end of the cycle for both groups (LOW_D *k* = 0.184 to 0.099; HIGH_D *k* = 0.146 to 0.079). The greater growth performances in LOW_D could be related to a lower competitive feeding behavior compared to the HIGH_D. In fact, a space of 60 cm for each animal is appropriate when feed is provided *ad libitum* (16). In the present study, the pen design allowed a maximum of 10 animals to feed simultaneously. However, LOW_D pens housed 7 animals, whereas HIGH_D pens housed 13 animals, potentially increasing competition for feed access in the latter. In addition to feeding behavior, increased space allowance may facilitate greater voluntary movement, thereby musculoskeletal development (22). Consistent with this hypothesis, carcass yield was greater in the LOW_D group compared with HIGH_D (58.89% vs. 57.96%, respectively) in agreement with previous findings reported by Ha et al., (2018).

Stressful conditions are known to affect several aspects of biological functioning, including immune competence (4). In adult cattle, lymphocytes represent the main component of the white blood cells with a neutrophil-to-lymphocyte ratio of 1:2. A stress leukogram is usually characterized by neutrophilia and lymphocytopenia, and occasionally monocytosis (24, 25). However, previous studies had reported no evidence of stress leukogram in cattle housed at space allowance below 6 m^2^/animal (4, 5, 23). Although transient stress responses have been observed during the early post-housing period at lower (1.2 to 4.2 m^2^/animal) space allowances (26). In this study, WBC, NEU, LYM, and MON were within reference range in both groups (25, 27, 28). Despite the absence of pathological conditions, the HIGH_D group had an increase in NEU and MON and a reduction in LYM from approximately mid-cycle production until the end of the study. In contrast, LOW_D had no changes in NEU and LYM over time, while MON reduced progressively. This condition may be indicative of slight greater stress in high-density animals, consistent with potentially greater competitive feeding behavior, even though all white cell populations were still within physiological ranges.

Regarding ultrasound evaluation, two parameters assessed fat composition (BFT and IMF) and two assessed muscle growth (DEPTH and RIBEYE). It has been reported that BFT is not affected by stocking density across a wide range of space allowances, including comparisons between 2–2.5 and 10–13 m^2^/animal, between 10 and 17 m^2^/animal, and between 8 and 32 m^2^/animal (22, 29, 30). Despite this, BFT is reported to respond positively to periods of early and late fattening when concentrate levels are usually increased (22). The BFT evaluated in this study increased progressively throughout the production cycle in both groups, likely in response to the animals finishing. However, the LOW_D showed a greater increase from the middle of the cycle (from D60 to D120). This result could also be related to potentially more competitive feeding behavior in the high-density group. Instead, IMF deposition occurs mainly at the end of the production cycle, with a greater increase in animals with lower density (17 vs. 10 m^2^/animal) (22). However, stressful conditions can affect the animals’ feed intake and increase fat breakdown (29). The beef heifers of this study had a progressive increase in IMF during the production cycle, with a greater level in LOW_D at D90 and D120 compared to the HIGH_D group. This result was consistent with the normal growth and fat deposition in beef animals and may further indicate a slightly greater stress as stocking density increases.

Muscle assessment of the *longissimus dorsi* using ultrasonography should include both longitudinal (DEPTH) and transverse (RIBEYE) measurements, as these parameters have a suboptimal correlation (*r* = 0.56) and therefore provide complementary information. The lack of both measurements results in a loss of information that could also be useful for the genetic improvement of herds (9). However, the only article to the best of the authors’ knowledge that also evaluated DEPTH according to stocking density found no differences between 10–13 and 2–2.5 m^2^/animal (29). On the contrary, the RIBEYE showed an increase as the space allowance per animal increased (22, 29, 30). In the current study, the LOW_D heifers had a greater DEPTH and RIBEYE from D30 to the end of the study further suggesting that an increased space allowance per animal can positively influence the musculoskeletal system development and improving muscle quantity.

This study was conducted at one commercial farm over two consecutive years (experimental trials). Although a uniform distribution of animals’ enrollment that exceeded the minimum sample size in both evaluation years, the balanced seasonal effect, and the inclusion of the boxes in the statistical analysis, a potential effect on the results cannot be entirely ruled out. Moreover, an individual feeding behavior with an evaluation of dry matter intake was not possible in a commercial farm representing a potential limitation of this study.

## 5 Conclusion

This study demonstrated that stocking density significantly affects growth performance and carcass traits in beef heifer. Animals housed at lower density (10.3 m^2^/animal) exhibited greater ADG, especially after the mid-cycle, higher final BW, and improved carcass yield compared with heifers housed at higher density (5.54 m^2^/animal). Increased space allowance was also associated with enhanced fat deposition and muscle growth, as assessed by ultrasonography, suggesting more favorable nutrient utilization and musculoskeletal development under low-density conditions. Although white blood cell counts remained within physiological refence ranges in both groups, heifers housed at higher density showed temporal increases in neutrophils and monocytes, accompanied by reductions in lymphocytes, indicating a mild but persistent stress response probably related to competitive feeding behavior. Overall, these findings highlight the importance of stocking density as a management factor affecting productivity and animal welfare in beef production systems. Providing a greater space allowance per animal, beyond minimum kinematic thresholds, appears to promote improved, growth performance, carcass yield, and better overall health.

## Data Availability

The original contributions presented in the study are included in the article/Supplementary material, further inquiries can be directed to the corresponding author.

## Footnotes

1
http://www.gpower.hhu.de/

2
https://www.R-project.org/
