# Supplementary material for: Impact of stocking density on beef heifer productive performance and health through hematology and longissimus dorsi ultrasound
Source: Front Vet Sci. 2026 Jun 3;13:1826548. doi: 10.3389/fvets.2026.1826548 (PMC13272476; doi:10.3389/fvets.2026.1826548)
Supplement: Supplementary file 1 [file Table_1.DOCX]

Supplementary Material

**Supplementary Table 1.** Values of Shapiro-Wilk test on evaluated parameters with p-values > 0.05.

| **Parameters** | ***Shapiro-Wilk*** |
| --- | --- |
| BW_D0,_ kg | 0.974 |
| BW_D60,_ kg | 0.931 |
| BW_D120,_ kg | 0.933 |
| ADG_D60_D0,_ kg/d | 0.971 |
| ADG_D120-D60,_ kg/d | 0.937 |
| ADG_D120-D0,_ kg/d | 0.919 |
| Carcass yield, % | 0.939 |
| RBC, ×10^6^ cells/µL | 0.993 |
| HGB, g/dL | 0.995 |
| HCT, % | 0.995 |
| MCV, fL | 0.973 |
| MCHC, g/dL | 0.991 |
| PLT, ×10^3^ cells/µL | 0.975 |
| WBC, ×10^3^ cells/µL | 0.949 |
| EOS, % | 0.977 |
| BAS, % | 0.919 |
| NEU, % | 0.914 |
| LYM, % | 0.934 |
| MON, % | 0.980 |
| LUC, % | 0.926 |
| DEPTH, cm | 0.987 |
| RIBEYE, cm^2^ | 0.977 |
| BFT, cm | 0.988 |
| IMF, % | 0.974 |

**Supplementary Table 2.** Results of the post-hoc power analysis performed on significant results.

| **Parameters** | **Post-hoc power** | **Parameters** | **Post-hoc power** | **Parameters** | **Post-hoc power** |
| --- | --- | --- | --- | --- | --- |
| BW_D120_, kg | 100% | NEU, % |  | DEPTH, cm |  |
| ADG_D120_D60_, kg/d | 100% | D0 | 83.3% | D0 | 92.9% |
| ADG_D120_D0_, kg/d | 83.8% | D30 | 10.4% | D30 | 100% |
| Carcass yield, % | 91.8% | D60 | 100% | D60 | 100% |
|  |  | D90 | 100% | D90 | 100% |
|  |  | D120 | 100% | D120 | 100% |
|  |  | LYM, % |  | RIBEYE, cm^2^ |  |
|  |  | D0 | 96.9% | D0 | 58.0% |
|  |  | D30 | 27.2% | D30 | 100% |
|  |  | D60 | 100% | D60 | 100% |
|  |  | D90 | 100% | D90 | 100% |
|  |  | D120 | 100% | D120 | 100% |
|  |  | MON, % |  | BFT, cm |  |
|  |  | D0 | 67.1% | D0 | 100% |
|  |  | D30 | 22.5% | D30 | 100% |
|  |  | D60 | 99.8% | D60 | 100% |
|  |  | D90 | 100% | D90 | 100% |
|  |  | D120 | 100% | D120 | 100% |
|  |  |  |  | IMF, % |  |
|  |  |  |  | D0 | 43.7% |
|  |  |  |  | D30 | 94.9% |
|  |  |  |  | D60 | 99.9% |
|  |  |  |  | D90 | 100% |
|  |  |  |  | D120 | 100% |

**Supplementary Table 3.** Not-significant parameters of the complete blood count (red blood cell (RBC), haemoglobin (HGB), haematocrit (HCT), medium corpuscular volume (MCV), mean corpuscular haemoglobin concentration (MCHC), platelets (PLT), white blood cell (WBC), eosinophils (EOS), basophils (BAS), and large unstained cells (LUC)) according to stocking density (LOW_D group, 10.3 m^2^/animal; HIGH_D group, 5.54 m^2^/animal), and time (D0, D30, D60, D90, D120).

| **Parameters** | **LOW_D (N=28)** | **HIGH_D (N=26)** | **SEM** | ***p-values^1^*** |
| --- | --- | --- | --- | --- |
| RBC, ×10^6^ cells/µL | | | | |
| D0 | 9.23 | 9.80 | 0.17 | G = 0.851  t < 0.001  G×t = 0.135 |
| D30 | 8.82 | 8.98 | 0.17 |  |
| D60 | 8.85 | 9.02 | 0.17 |  |
| D90 | 9.02 | 9.17 | 0.17 |  |
| D120 | 8.57 | 8.68 | 0.17 |  |
| HGB, g/dL | | | | |
| D0 | 13.7 | 14.1 | 0.24 | G = 0.407  t = 0.319  G×t = 0.451 |
| D30 | 13.0 | 12.9 | 0.24 |  |
| D60 | 13.5 | 13.6 | 0.24 |  |
| D90 | 14.0 | 14.2 | 0.24 |  |
| D120 | 13.8 | 13.8 | 0.24 |  |
| HCT, % | | | | |
| D0 | 36.1 | 36.0 | 0.64 | G = 0.102  t = 0.137  G×t = 0.114 |
| D30 | 35.5 | 35.8 | 0.64 |  |
| D60 | 35.7 | 35.7 | 0.64 |  |
| D90 | 36.9 | 36.9 | 0.64 |  |
| D120 | 36.1 | 36.1 | 0.64 |  |
| MCV, fL | | | | |
| D0 | 38.3 | 38.8 | 0.55 | G = 0.191  t = 0.022  G×t = 0.871 |
| D30 | 39.4 | 39.8 | 0.55 |  |
| D60 | 40.5 | 39.6 | 0.56 |  |
| D90 | 41.1 | 40.3 | 0.56 |  |
| D120 | 41.4 | 41.6 | 0.56 |  |
| MCHC, g/dL | | | | |
| D0 | 36.9 | 37.2 | 0.18 | G = 0.130  t < 0.001  G×t = 0.268 |
| D30 | 36.9 | 37.0 | 0.18 |  |
| D60 | 37.7 | 38.1 | 0.18 |  |
| D90 | 38.0 | 38.5 | 0.18 |  |
| D120 | 38.2 | 38.1 | 0.18 |  |
| PLT, ×10^3^ cells/µL | | | | |
| D0 | 314 | 343 | 19.1 | G = 0.157  t = 0.623  G×t = 0.745 |
| D30 | 341 | 356 | 19.3 |  |
| D60 | 321 | 325 | 19.1 |  |
| D90 | 323 | 352 | 19.3 |  |
| D120 | 304 | 349 | 19.3 |  |
| WBC, ×10^3^ cells/µL | | | | |
| D0 | 7.95 | 8.28 | 0.30 | G = 0.792  t = 0.282  G×t = 0.317 |
| D30 | 8.44 | 8.21 | 0.30 |  |
| D60 | 8.10 | 8.38 | 0.30 |  |
| D90 | 8.12 | 8.50 | 0.30 |  |
| D120 | 8.09 | 8.87 | 0.30 |  |
| EOS, % |  |  |  |  |
| D0 | 2.51 | 2.44 | 0.56 | G = 0.356  t < 0.001  G×t = 0.417 |
| D30 | 2.71 | 2.36 | 0.56 |  |
| D60 | 2.89 | 3.41 | 0.56 |  |
| D90 | 3.68 | 3.80 | 0.56 |  |
| D120 | 4.49 | 3.96 | 0.56 |  |
| BAS, % |  |  |  |  |
| D0 | 1.12 | 1.15 | 0.06 | G = 0.344  t = 0.008  G×t = 0.388 |
| D30 | 1.18 | 1.04 | 0.06 |  |
| D60 | 1.06 | 1.02 | 0.06 |  |
| D90 | 1.04 | 1.03 | 0.06 |  |
| D120 | 1.01 | 0.96 | 0.06 |  |
| LUC, % |  |  |  |  |
| D0 | 0.63 | 0.61 | 0.04 | G = 0.233  t < 0.001  G×t = 0.489 |
| D30 | 0.40 | 0.42 | 0.04 |  |
| D60 | 0.33 | 0.29 | 0.04 |  |
| D90 | 0.35 | 0.42 | 0.04 |  |
| D120 | 0.66 | 0.72 | 0.04 |  |
| ^1^ G or Group effect; t or time effect; G×t or Group*time effect. | | | | |
